# Digital technologies used in clinical trial recruitment and enrollment including application to trial diversity and inclusion: A systematic review

**DOI:** 10.1177/20552076241242390

**Published:** 2024-03-28

**Authors:** Amy Kasahara, Jennifer Mitchell, Joshua Yang, Raphael E. Cuomo, Tiana J. McMann, Tim K. Mackey

**Affiliations:** 1 90621 Rady School of Management, University of California San Diego, La Jolla, CA, USA; 2 Department of Public Health, University of California Irvine, Irvine, CA, USA; 3 Occupational Therapy, California State University Dominguez Hills, Carson, CA, USA; 4 Department of Public Health, California State University Fullerton, Fullerton, CA, USA; 5 Global Health Policy and Data Institute, San Diego, CA, USA; 6 S-3 Research, San Diego, CA, USA; 7 Department of Anesthesiology, University of California San Diego – School of Medicine, San Diego, CA, USA; 8 Global Health Program, Department of Anthropology, 8784 University of California San Diego, San Diego, CA, USA

**Keywords:** COVID-19, digital health technology, clinical trials

## Abstract

**Background:**

Many clinical trials fail because of poor recruitment and enrollment which can directly impact the success of biomedical and clinical research outcomes. Options to leverage digital technology for improving clinical trial management are expansive, with potential benefits for improving access to clinical trials, encouraging trial diversity and inclusion, and potential cost-savings through enhanced efficiency.

**Objectives:**

This systematic review has two key aims: (1) identify and describe the digital technologies applied in clinical trial recruitment and enrollment and (2) evaluate evidence of these technologies addressing the recruitment and enrollment of racial and ethnic minority groups.

**Methods:**

We conducted a cross-disciplinary review of articles from PubMed, IEEE Xplore, and ACM Digital Library, published in English between January 2012 and July 2022, using MeSH terms and keywords for digital health, clinical trials, and recruitment and enrollment. Articles unrelated to technology in the recruitment/enrollment process or those discussing recruitment/enrollment without technology aspects were excluded.

**Results:**

The review returned 614 results, with 21 articles (four reviews and 17 original research articles) deemed suitable for inclusion after screening and full-text review. To address the first objective, various digital technologies were identified and characterized, which included articles with more than one technology subcategory including (a) multimedia presentations (19%, *n* = 4); (b) mobile applications (14%, *n* = 3); (c) social media platforms (29%, *n* = 6); (d) machine learning and computer algorithms (19%, *n* = 4); (e) e-consenting (24%, *n* = 5); (f) blockchain (5%, *n* = 1); (g) web-based programs (24%, *n* = 5); and (h) virtual messaging (24%, *n* = 5). Additionally, subthemes, including specific diseases or conditions addressed, privacy and regulatory concerns, cost/benefit analyses, and ethnic and minority recruitment considerations, were identified and discussed. Limited research was found to support a particular technology's effectiveness in racial and ethnic minority recruitment and enrollment.

**Conclusion:**

Results from this review illustrate that several types of technology are currently being explored and utilized in clinical trial recruitment and enrollment stages. However, evidence supporting the use of digital technologies is varied and requires further research and evaluation to identify the most valuable opportunities for encouraging diversity in clinical trial recruitment and enrollment practices.

## Introduction

Many clinical trials fail due to poor recruitment and enrollment with nearly 86% of all trials failing to meet enrollment goals and 32% of Phase III trials failing because of recruitment-related issues.^1–4^ Within cancer research, less than 5% of patients are enrolled in clinical trials due to several issues that could be partly addressed through the utilization of technology.^5,6^ Some of these barriers include limited resources, lack of awareness about available trials, geographical issues, and attitudinal concerns.^6^ The financial cost of a failed clinical trial can be $800M to $1.4B and can lead to delays in the development of potentially lifesaving therapies.^1,7^ Early clinical trial termination due to recruitment issues further impacts subsequent research by reducing patient trust and future willingness to participate in clinical trials.^8^

Additionally, the lack of inclusion of difficult-to-reach participants in clinical trials, including racial and ethnic minorities, can lead to poor quality studies or limit the external validity of results and hinder trials’ ability to meet enrollment milestones.^9,10^ For example, certain racial and ethnic minority groups suffered from increased morbidity and mortality during the COVID-19 pandemic, yet were underrepresented in COVID-19 clinical trial recruitment and enrollment. While Black communities represent 13% of the U.S. population, they accounted for 21% of COVID-19 mortality and only 3% of COVID-19 vaccine clinical trial participants.^11^ Similar mortality and clinical trial representation data have been reported for other minority groups in the United States.^12^ These health disparities have led to regulatory agencies issuing guidance to develop and adopt plans for industry-sponsored trials to enroll more diverse populations.^13^

With the continued “digitization” of the healthcare industry towards increasing adoption of digital tools across the healthcare delivery and value ecosystem, the opportunity to leverage innovative technologies to improve the management of clinical trials has grown. Digital technology should allow for investigators to reach a large number of participants not previously accessible for clinical trials, as there are around 4.54B internet users, 307.3M users in the United States alone.^14,15^ The use of digital technology may also reduce costs associated with clinical trial management, including those associated with labor and timely manual processes, and improve staff diversity which is linked to increased engagement from minority populations.^16^ Hence, solutions to improve both the efficiency and diversity of clinical trial recruitment and enrollment are paramount to the future of addressing populations most impacted by disease and implementing rapid public health interventions.

Past studies examining the use of digital technologies for clinical trial recruitment and enrollment have limited their scope to the use of social media as a means to improve recruitment outcomes.^17^ Therefore, a greater understanding of the current utilization of all types of digital technologies in the enrollment and recruitment phases of clinical trials will help identify challenges and opportunities to address in future research. Additionally, promoting solutions to engage diverse populations in clinical trials is essential for the continued progression of research and development of biomedical therapeutics that address long-standing health equity issues.

### Statement of objective/aims of the review

The aims of this systematic review are twofold: (1) to identify and characterize digital technologies used for clinical trial recruitment and enrollment and (2) to assess if there is evidence that these technologies can increase ethnic and racial minority recruitment and enrollment. Hence, the aim of this systematic review was to explore existing research on the use of digital technologies in the recruitment and enrollment stages of clinical trials. Identification of sub-themes within the literature was also conducted to identify challenges and opportunities for further exploration and additional research.

## Methods

This review provides a systematic synthesis of studies that specifically focus on digital technologies to promote enrollment and recruitment in clinical trials. We define “digital technology” as concepts, tools, and solutions that utilize internet-based information and communication systems, including online portals and management systems, cloud-based platforms, social networking sites, mobile and wireless devices, and electronic databases.

In the initial stages of this research, the decision to conduct a systematic review emerged organically from an evolving understanding of the literature and its gaps. As such, the review was not prospectively registered in a systematic review registry. To mitigate potential biases and to maintain rigor in our systematic review, we adhered strictly to the Preferred Reporting Items for Systematic Reviews and Meta-Analyses (PRISMA) guidelines throughout our research process. All steps outlined in the PRISMA checklist, from literature search to data extraction and analysis, were documented in detail to ensure reproducibility and transparency.

**Figure 1.**
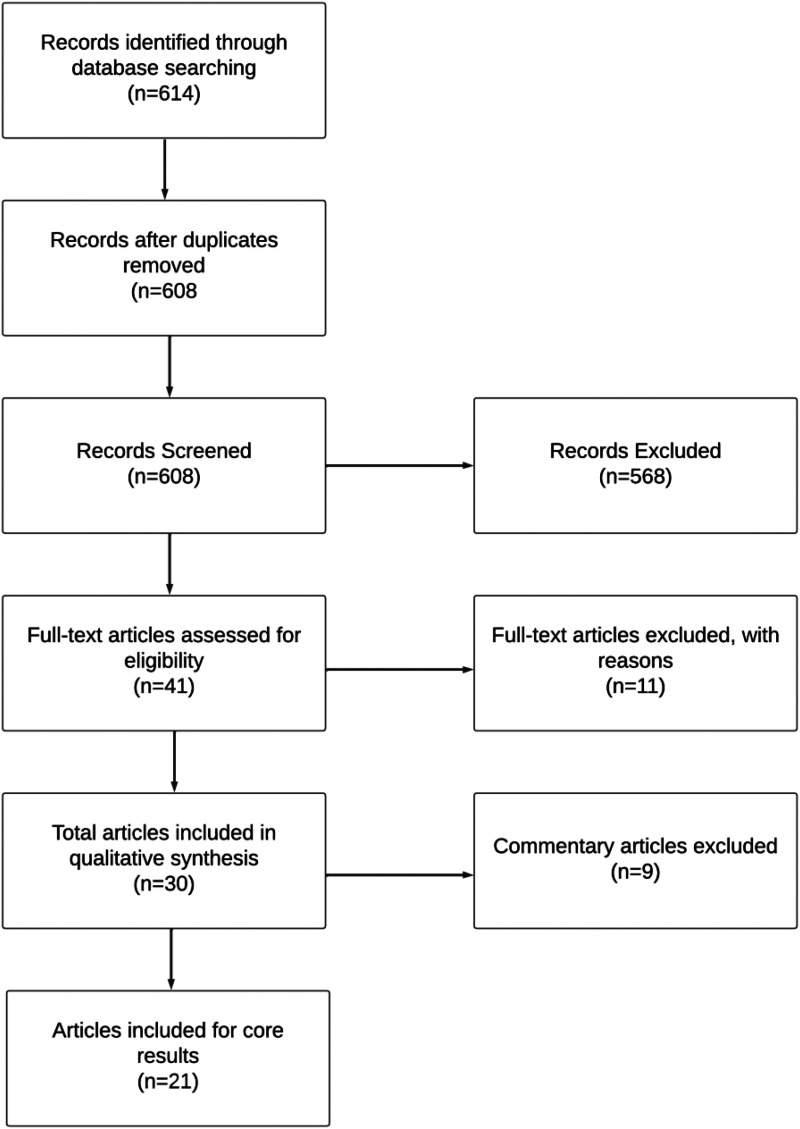
PRISMA flowchart.

In accordance with PRISMA guidelines, we carried out an interdisciplinary review in July 2022. Specifically, we reviewed journal articles, original research, conference papers, case reports, and technology reviews that were indexed in three scholarly databases: PubMed (Medline), IEEE Xplore, and ACM Digital Library. We selected these databases based on the interdisciplinary nature of the study aims which required a review of the science/health literature (from PubMed-indexed journals that cover life sciences and biomedical topics); studies on information, communication, and engineering technologies (from IEEE Xplore-indexed articles that focus on scientific and technical content published by the IEEE); and research on advances in computing sciences (from ACM Digital Library, which indexes various journals, conference proceedings, technical magazines, newsletters and books in the computing literature). See Figure 1 for an overview of the PRISMA flowchart and Supplemental File for additional PRISMA methods.

PRISMA: Preferred Reporting Items for Systematic Reviews and Meta-Analyses.

The three databases were searched for articles published in the English language between January 2012 and July 2022, using the Medical Subject Headings (MeSH) unique ID terms “Clinical Trials” (ID: D002986) and “Technology” (ID: D013672). Other non-MeSH key search terms included “Enroll*,” “Recruit*,” and “Digital.” Keywords were queried in the Title/Abstract field using the advanced search function settings for PubMed, IEEE Xplore, and ACM Digital Library databases (see Supplemental File for additional details). We chose a 10-year literature review period as the aims of this review focused on relatively new or innovative technologies. Finally, we also include findings of themes generated by the nonoriginal articles (e.g., opinion, commentary, viewpoints, etc.) in the discussion section of this article that were not included in the main results of this review as we wanted to further explore themes for racial and ethnic minority trial inclusion, as well as topics related to cost-effectiveness, ethics, and regulatory considerations that may be discussed in these content types.

## Results

A total of 614 results were reviewed from searches conducted on PubMed, IEEE Xplore, and ACM Digital Library. After the initial screening process, which involved removing duplicates and reviewing titles/abstracts, a full-text review of 41 articles was conducted for relevance to the study inclusion and exclusion criteria. Eleven articles^6,18–27^ were excluded following a full-text review that identified them as not meeting inclusion criteria. Any disagreement related to inclusion between primary and secondary researchers (AH and JM) was resolved through discussion with a third researcher and senior author (TKM). Subsequently, 21 articles were deemed eligible for inclusion in the main results, 21 from Pubmed, 0 from IEEE, and 0 from ACM. Types of articles included research articles (81%, *n* = 17) and reviews (19%, *n* = 4) based in five different countries, with the majority from the United States (81%, *n* = 17). See Table 1 for geography, year of publication, and disease studied and Supplemental File for a full list of articles selected for inclusion.

**Table 1.** Geography, technology type, year of publication, and disease type.

Theme	Subcategory	No of articles	% of total^a^
Technology type (totals >100% due to multiple subcategories per article)			
	Blockchain	1	5
	Mobile apps	3	14
	Machine learning/EHR	4	19
	Multimedia	4	19
	Web platform	5	24
	E-consent	5	24
	Virtual messaging	5	24
	Social media	16	29
Geography			
	United States	17	81
	Canada	1	5
	England	1	5
	Germany	1	5
	Switzerland	1	5
Year of publication			
	2012	2	10
	2013	2	10
	2014	1	5
	2015	2	10
	2016	0	0
	2017	1	5
	2018	1	5
	2019	2	7
	2020	1	13
	2021	5	17
	2022^a^ (partial year)	4	19
Diseases studied			
	Cancer	7	33
	Cardiovascular	1	5
	COVID19	1	5
	Lifestyle/risk factors	3	14
	Neuro	2	10
	Not specified	17	33

a Indicates number of articles that met inclusion criteria for this study but only for a partial year of 2022 (up to July 2022).

The majority (81%, *n* = 17) of reviewed publications were original research articles that included empirical evidence relating to recruitment and/or enrollment with the described digital technology (see Table 2 for summary or original research articles included in this study). The remaining publications (19%, *n* = 4) were reviews that did not present empirical data or analysis. Only four original research studies were randomized controlled trials (RCTs), with the remaining comprised of observational and feasibility studies. The technology types for the RCTs included the use of web-based materials, namely, QR codes,^28^ multimedia technology through the use of an interactive iPad program,^29^ virtual messaging via optimized emails,^30^ and machine learning to automate eligibility notification, delivery of research materials, and informed consent^31^ While outcomes across studies were not uniform, all found positive results through the use of digital technology during recruitment and/or enrollment (see Supplemental File for additional RCT findings).

**Table 2.** Summary of original research articles included in this study.

Title	Authors	Year	Type of intervention	Size of study	Category of tech	Disease category	Geography	Main finding
Assessment of automated clinical trial recruitment and enrolment using patient-facing technology	Bardach, N	2021	RCT	1012 patients	Machine learning	Not specified	United States	Interactive patient care systems (IPCS) streamlines study implementation tasks like randomization and collecting e-consent and contact information at the bedside, it does not demonstrate recruitment efficiencies.
Automated real-time text messaging as a means for rapidly identifying acute stroke patients for clinical trials	Jegzentis, K	2014	Observational	513 text messages	Virtual messaging, machine learning	Neurological	Germany	The text messaging system utilizing IT-based Clinical Information Systems (CIS) was able to identify acute stroke trial patients, documented routinely, and did not result in higher workload.
Deployment of an end-to-end remote, digitalized protocol in COVID-19: process evaluation	Zahradka, N	2022	Observational	13 study advertisements, 38 eligibility forms	Social media, e-consent	COVID-19	United States	Evaluating clinical study implementation is crucial; effective online advertising and enrollment can lower costs, wearable technology provides detailed monitoring.
Development of a digital research assistant for the management of patients’ enrollment in oncology clinical trials within a research hospital	Cesario A	2021	Observational	96 patients	Mobile applications, machine learning	Cancer	Switzerland	The Digital Research Assistant proved to be an effective tool for patients’ data sharing within the single institution and setting up networks with other cancer centers. The platform can be useful as a valid research tool supporting clinicians and scientists working in high and low volume centers.
Digital recruitment and enrollment in a remote nationwide trial of screening for undiagnosed atrial fibrillation: lessons from the randomized, controlled mSToPS trial	Baca-Motes K	2019	RCT	2659 participants	Virtual messaging	Cardiovascular	United States	Improvements in the recruitment campaign emphasizing new technology in a five-step outreach, boosted enrollment rates from 0.8% to 9.4% in a large remote clinical trial, showcasing the potential of targeted strategies while underscoring the need for ongoing research on recruitment and retention.
Efficacy and cost-effectiveness of an automated screening algorithm in an inpatient clinical trial	Beauharnais, C	2012	Observational	2698 patients	Machine Learning	Lifestyle/ Risk Factors	United States	Use of a screening algorithm decreased screening time from 4 to 2 h and increased average number of patients prescreened per day (P < .0001). The algorithm was cost-neutral after enrolling 12 patients.
Facebook recruitment for children with advanced cancer and their parents: lessons from a web-based pediatric palliative intervention study	Cho E	2021	Observational	150 child–parent dyads	Social media	Cancer	United States	Researchers used Facebook advertisements to recruit children for a web-based intervention and were able to enroll 150 dyads for participation.
Improving the patient–clinician interface of clinical trials through health informatics technologies	Carrion, J	2018	Review	N/A	Social media, machine learning, mobile applications	Not specified	United States	This review focused on technology-driven improvements for the patient–clinical trial interface, specifically for explanatory pharmaceutical trials.
Innovating information-delivery for potential clinical trial participants. What do patients want from multimedia resources?	Shneerson, C	2013	Observational	72 participants	Multimedia, web platform	Not specified	England	A questionnaire was delivered to 72 participants from cancer support groups to determine views on the design and use of multimedia resources to deliver clinical trial info. Multimedia resources were viewed positively.
Responding to a significant recruitment challenge within three nationwide psychoeducational trials for cancer patients	Stanton, A	2013	Observational	2134 participants	Virtual messaging, web program	Cancer	United States	Over a 33-month period recruitment was tracked for three nationwide psychoeducational trials for cancer patients. The majority of patients were recruited using information programs and registries.
Using digital multimedia to improve parents’ and children's understanding of clinical trials.	Tait AR	2015	RCT	148 parent participants and 135 child participants	Multimedia	Not specified	United States	Participants were randomized to receive information regarding clinical trials using a traditional paper format or interactive iPad program. Children in the iPad group had a significantly greater understanding than children in the paper format group. The iPad format was found to be significantly easier to follow and more effective.
Clinical trial management of participant recruitment, enrollment, engagement, and retention in the SMART study using a Marketing and Information Technology (MARKIT) model	Gupta A	2015	RCT	404 participants	Social media, virtual messaging, web platform	Lifestyle/risk factors	United States	Digital and print media were both used to drive traffic to the study recruitment website. A total of 11,864 page visits by 7762 unique visitors resulting in 1941 interested visitors that completed an online interest form. Direct traffic (likely from emails and printed material distribution) accounted for 59% of site traffic, with 14% coming from search engines and 27% from Facebook and other websites.
Association of remote technology use and other decentralization tools with patient likelihood to enroll in cancer clinical trials	Adams, D	2022	Observational	1183 respondents	E-consenting, mobile applications	Cancer	United States	Participants participated in a cross-sectional survey study for survivors of cancer. Results demonstrated an increased likelihood toward participation in trials where the use of remote technology and other decentralization tools would reduce the need for travel to a trial site.
Digital health applications for pharmacogenetic clinical trials.	Naik H	2020	Review	N/A	Web platform, e-consent, machine learning	Not specified	United States	This article explored the various uses of digital health technologies in pharmacogenetic trials.
Social media use for research participant recruitment: integrative literature review	Darko, E	2022	Review	96 records	Social media	Not specified	Canada	Findings from the review demonstrate that social media is a suitable, viable, and cost-effective option for recruitment of research participants.
A pilot study of a culturally targeted video intervention to increase participation of African American patients in cancer clinical trials	Banda, D	2012	Pilot Study	108 patients	Multimedia presentation	Cancer	United States	A 15 min culturally targeted video was designed to impact six specific attitudes of African American cancer patients toward therapeutic trials. Results showed a positive change in intention for 36% of the participants.
Investigation of a multimedia, computer-based approach to improve knowledge, attitudes, self-efficacy, and receptivity to cancer clinical trials among newly diagnosed patients with diverse health literacy skills	Polite, B	2019	Observational	120 patients	Multimedia	Cancer	United States	An interactive teaching video was used to include patients’ first oncology appointment. Surveys were provided before and after and demonstrated improvements in participants’ knowledge, positive attitudes and self-efficacy.
Processes in increasing participation of African American women in cancer prevention trials: development and pretesting of an audio-card	Kenerson, D	2017	RCT	60 participants	Multimedia	Cancer	United States	An audio-card using cultural and linguistic elements was provided to 30 participants and compared to 30 controls to determine cancer prevention trial beliefs and behavioral intent. There was a statistically significant difference in personal value, social influence, and personal barriers for the treatment group compared to the control group.
Recruiting young women of color into a pilot RCT targeting sexual health: lessons learned and implications for applied health technology research	Gonzalez, S	2022	Observational	114 participants	Social media, virtual messaging	Lifestyle/risk factors	United States	Banner ads on Facebook and OkCupid as well as targeted electronic outreach (through emails to community-based organizations and professors at local colleges) were used to recruit participants. Greater enrollment occurred through the targeted electronic recruitment method than through banner ads.
Decentralized clinical trials	Van Norman, G.	2021	Review	N/A	Web platform, blockchain	Not specified	United States	This review explored the advantages and challenges for decentralized clinical trials.
General practice and digital methods to recruit stroke survivors to a clinical mobility study: comparative analysis	Reuter, K	2021	Observational	40 participants	Social media, web platform	Neurological	United States	The study was promoted digitally using Facebook and Google AdWords with a total of eight advertised messages over 5 weeks resulting in nine participants, while traditional recruitment took 54 weeks and resulted in 31 participants.

Identified literature discussing the use of digital tools in clinical trial enrollment and recruitment ranged in diseases studied, including cardiovascular disorders (5%, *n* = 1), COVID-19 (5%, *n* = 1), lifestyle/risk factors (14%, *n* = 3), and neurological disorders (10%, *n* = 2). Cancer was the most common category of diseases studied (33%, *n* = 7). The recent COVID-19 pandemic was also mentioned in several articles as a reason for the increased adoption of digital health in clinical trials. However, there was only one COVID-19-specific article identified in the search parameters of this study which explored an end-to-end digitalized clinical study protocol but was limited to a small sample size of eight subjects, despite using social media (Facebook ads) as their recruitment platform. The small number of studies^32^ related to COVID-19 was likely influenced by our study time period. None of the articles included discussed the use of digital technologies specifically in a drug development clinical trial.

The comparison of costs and benefits was often addressed in the literature in the context of digital tools. Many articles suggested that the use of digital technologies could reduce costs related to recruitment and enrollment.^3,9,30,33,34^ However, overall there are inconsistent findings regarding the financial benefit of the use of digital technology for enrollment and recruitment, with some digital technologies (e.g. email distribution) being viewed as more cost-effective to reach large populations when compared to other technologies (e.g. social media ads). See Supplemental File for additional cost/benefit results.

In the subsequent sections, we discuss details of the specific digital technology types identified, whether technology specifically addressed health equity or ethnic and minority topics, and policy and regulatory considerations raised in the literature.

### Technology types

The major technology types identified in these studies were grouped as follows: (a) multimedia presentations (i.e. PowerPoint, educational videos); (b) mobile applications; (c) social media platforms (e.g., Facebook); (d) machine learning and computer algorithms; (e) e-consenting tools; (f) distributed ledger and blockchain technologies; (g) web-based programs; and (h) virtual messaging. Social media, virtual messaging, web-based platforms, and machine learning/electronic health record (EHR) were the most frequently discussed and evaluated digital technologies, while multimedia, mobile applications and blockchain were less frequently explored or formally evaluated. To characterize findings, we grouped technologies into categories based on similar characteristics (see Table 3 for technology type descriptions). It should be noted that many of these groups could be further subcategorized into other technology types or specific modalities (e.g., “virtual messaging” included both email and text messaging).

**Table 3.** Technology type used in clinical trial recruitment and enrollment.

Technology type	Description of technology	Application to clinical trial recruitment/enrollment
Multi-media presentation	Information presented with slides, videos, or websites that are useful for different forms of digital communication	Video education of study protocol
Mobile applications	Digital technology for use on small, wireless devices such as a tablet or smartphone	Clinical trial management software
Social media use	Websites and applications that enable users to create and share content or to participate in social networking	Targeted recruitment advertisements
Machine learning/computer algorithms/EHR data mining	Use of machine learning, computer algorithms and/or electronic health record data mining	Enhancing enrollment by reducing heterogeneity, choosing patients more likely to have a measurable clinical endpoint, and identifying populations more capable of responding to treatment Identifying potentially eligible participants by prescreening patients for demographics and preexisting health conditions
Electronic consent	Electronic systems to communicate with participants about trial information and to obtain documented informed consent	Informed consent through virtual appointment
Distributed ledger technology/blockchain	Linked-list structure distributed over a peer-to-peer network that provides a systematic approach to maintain the order of transactions throughout a peer-to-peer network	Clinical trial management system HIPAA-compliant data exchange Automated e-consent process
Web-based programs	Multimedia, multichannel, internet facilitated programs	Increase in accuracy and precision of clinical trial data vs. traditional documentation during recruitment/enrollment eligibility and intake
Virtual messaging	Technology-based individual communications, primarily in formats such as email or SMS messaging	Email recruitment campaign

#### Multimedia presentation

Multimedia presentations include information presented with slides, videos, or websites that are useful for different forms of digital communication. Multiple articles reviewed the use of multimedia presentations for educational purposes, in particular during the recruitment and consenting phases of clinical trials. Overall, participants were purportedly able to achieve greater comprehension of the research protocol with video consent because they could watch an instructional or informational video at their own pace. It was also noted that this could be helpful in populations where health literacy rates are low.

Several articles discussed the use of digital technology to enable online users to join communities by sharing their clinical trial experiences in forums. However, a related key barrier to clinical trial enrollment in the context of knowledge and information seeking was a lack of overall public awareness about clinical trials.

Interactive multimedia technology was also shown to be beneficial in learning. Shneerson et al. developed a questionnaire with quantitative questions rating on a four-point agreement scale and qualitative open-ended questions to determine which design features of multimedia tools were best for delivering clinical trial information.^35^ Individuals viewed multimedia resources that were easy to use and had visual aids as a positive way to learn about the trial. Multimedia was considered an enhanced delivery of information and helped participants have more control of their learning experience.

Another study described a clinical trial that evaluated the difference between using a traditional paper format trial education approach compared to an interactive iPad program in parents and children on their understanding of clinical trial concepts and participation to enroll.^29^ While there was no statistical difference in the post-test understanding of the clinical trial requirements between paper format and iPad group for parents, children in the iPad group had a significantly greater understanding than the children in the traditional paper group, indicating that different educational delivery modalities may differ in effectiveness depending on factors such as age and existing knowledge of clinical trials.

Audiocards were also used to educate participants about clinical trials in cancer prevention. An audio tool was developed and tested to address suboptimal participation of African-American women in cancer prevention trials.^36^ This tool incorporated cultural and linguistic elements, as well as visual messages, to share the importance of participation. Thirty African American women in the test group with audiocards were compared with 30 controls. Results indicated that there was a statistically significant increase from pre-test to post-test in social influence and personal values when compared to the control group, and also that health-related behavior was influenced by a person's social context. The higher score on post-test suggests that social influence may be a powerful motivator of behavior and attitudes toward clinical trials and that cultural values can provide a framework for tailoring messaging.

#### Mobile applications

Mobile applications are a recognizably growing area of digital technology that are designed specifically for use on small, wireless devices such as tablets or smartphones. While we were able to identify a few articles that specifically discussed unique mobile applications, several other well-known applications were discussed regularly but are more appropriately categorized as social media (e.g., Facebook) for the purposes of this review.

One study explored the use of a progressive web application in developing a digital trial research assistant.^5^ This tool uses software that can be used on multiple electronic devices (PC, tablet, smartphone, etc.) and acts as if it were a native app on the device on which it is being used. The application allowed for a number of operational functions: checking patient status, adding new patients, and viewing real-time updates. Patient characteristics were added to a database and then, using inclusion/exclusion criteria, algorithmic matchmaking occurred to link patients to a trial. This application was not specifically patient-facing and was instead targeted as a clinical trial management software, though was positioned as having the potential to improve communication among professionals running clinical trials.

#### Social media use

Social media was discussed frequently throughout the literature, primarily regarding its ability to reach a wider audience of potential trial participants through advertisements for recruitment. There are approximately 3.8B active social media users worldwide across popular social media platforms with various user adoption rates.^15^ Due to the general popularity and reach of Facebook as a mainstream social media platform, studies reported it as the most regularly used platform for trial recruitment and enrollment. The use of social media for targeted advertisements is often thought to improve costs related to recruitment, as well as promoting trial access to racial and ethnic minorities and potential participants in harder-to-reach geographic regions.

One study explored how Facebook could act as a recruitment platform through paid advertisements and discussed the potential value in the recruitment of difficult-to-access populations, such as vaccine-cautious parents, low-income women, immigrant healthcare providers, and transplant recipients.^10^ In this study, the investigator created a study Facebook page and customized advertisements that ran for 3 years (2015–2018). The study found that having interested persons answer brief screening questions before providing contact information was more effective than requesting contact information first.

The results of studies comparing social media-based recruitment strategies with those of traditional methods were mixed. Cho et al. (2021) references a review demonstrating improved recruitment using social media in only 12 out of 30 medical research studies. This review found that social media campaigns tended to recruit subjects who were female, white, non-Hispanic, and college-educated highlighting that outreach may be skewed with regards to race or other important demographics.^10^

Gonzalez and Grov explored the use of banner ads on Facebook and OkCupid (a dating app) for recruiting young women of color into a sexual health RCT.^37^ Several versions of banner ads were used, and this was compared to a more traditional outreach method (e.g., recruitment flyers sent to professors to forward to students and community-based organization emails). Facebook ads led to only two enrolled participants while OkCupid ads generated no participants. Overall, traditional electronic recruitment methods were deemed more cost-effective compared to those enrolled via Facebook. Among the banner ads tested, the ad with an image of a Black woman outperformed both the ad with the logo only and the ad with a Hispanic woman, indicating potential varying effectiveness for specific trial demographics.

Researchers conducted a COVID-19-based digital study which advertised on multiple social media platforms (Facebook, Instagram, LinkedIn).^32^ Despite having 8852 clicks on the advertisements, there were only 9 participants who enrolled in the study—although authors reported that the timing of the study (March 2021–May 2021) may have also contributed to poor enrollment, as COVID-19 rates were declining. The authors also cited a previous meta-analysis of traditional recruitment strategies that reported higher conversion rates than online recruitment.^38^

A systematic review, specifically for social media recruitment methods, concluded that the use of social media in clinical trials was growing, evidenced by the number of publications each year on the topic.^15^ They concluded that social media recruitment was more successful for noninterventional studies and recognized that Facebook was the most widely used application among researchers, likely due to the global penetration and popularity of the platform.

#### Machine learning/computer algorithms/EHR data mining

A few publications explored the applications of machine learning in clinical trials, including recruitment and enrollment specifically. One article showed an improved rate of enrollment from 0.17 to 0.32 patients per screening day when using an AI screening tool to review and identify candidates in an acute hospital setting.^39^

EHRs are helpful in identifying potential clinical trial participants by allowing for the screening of their demographics and health condition (age, disease, race/ethnicity, etc.) in order to match to trial opportunities. Studies like pharmacogenetic trials benefit from digital screening to determine patients who fit certain eligibility criteria and are more likely to be good responders to the study medications.^40^ Machine learning and deep learning can find patterns in large datasets of speech, images, or text to correlate diverse covariates within EHRs for improved patient-trial matching and recruitment. As patient recruitment accounts for one-third of the overall trial duration, the use of machine learning and data mining of EHRs was reported as having the potential to decrease the time required to identify suitable participants and accelerate trial completion.^1^

#### E-consenting

Electronic consenting (E-consenting) uses electronic systems to communicate with participants about trial information and to obtain documented informed consent. Though several types of trials involve consent forms and documentation processes which could be automated using prerecorded information, it was reported that pharmacogenetic trials are particularly complex and therefore may be best served by models involving digital automated consent.^40^

Informed consent through virtual appointment can potentially better ensure that the three requirements of information, comprehension, and voluntariness are met.^32^ In addition, e-consenting allows for the opportunity to enroll in a clinical trial that would otherwise not be feasible due to distance. In a study assessing the use of remote technology to reduce participation-related time and travel, 60–85% of respondents indicated they would be more likely to participate in a trial if they were able to use remote technology to decrease the need to travel.^41^ Hence, the authors concluded that leveraging remote technology and decentralization tools may increase patient consent rates and ultimately the presence of an e-consent tool may lead to better enrollment.

Web-based services, including e-consenting and clinical trial study webpages or landing pages were explored and are perhaps the most widely used category of digital technology available in the literature. Processes that use web-based programs, such as e-consenting, are reported as becoming a preferred method for clinical trial consenting due to reduced paperwork burden, decreased likelihood of lost forms, and improved security of digital platforms. Evidence suggests that the Internet is widely used as a primary source of information for health-related conditions, illustrating the potential for electronic resources (such as e-consent) to also improve knowledge of and participation in clinical trials.^35^

#### Distributed ledger technology/blockchain

Distributed ledger technology and blockchain technology, generally defined as a linked-list structure distributed over a peer-to-peer network that provides a systematic approach to maintain the order and provenance of transactions, was also discussed as a potential innovative and disruptive tool to enhance clinical trial enrollment and recruitment.^42^ With respect to clinical trials, blockchain has been proposed as a tool or IT infrastructure to better enable clinical trial management, automating and validating the e-consent process through cryptography, and allowing for the tracking, sharing and management of data used in clinical research.^43^

For example, a blockchain system called Quorum was discussed in one article to improve clinical trial management systems.^4^ Quorum purports to offer a better transaction throughput and lower latency compared with traditional blockchain technology applications. Of the 6000 patients used in this study, 1145 were matched in 1.39 s using 10 recruitment criteria using a matching algorithm on a smart contract, automated actions that can be coded and executed once a set of conditions are met rather than a contract designed in legal language.^42^ However, blockchain, as an emerging technology, faces technical challenges for use in healthcare such as scalability. Nevertheless, the enhanced ability to quickly recruit patients through smart contracts, and the potential for simplified enrollment via digital signatures, highlights the potential offerings of blockchain to address existing process inefficiencies within clinical trial management.

#### Web-based programs

It has also been reported that digital forms may lead to an increase in the accuracy and precision of clinical trial data.^34^ Traditional forms that require entry into research databases can demonstrate failure at both the participant as well as the staff or researcher level. Digitized forms lower this potential friction at both points by decreasing human error that results from the use of traditional documentation. They may reduce decision-making time and accuracy on enrollment appropriateness, prevent unnecessary delays from incomplete documentation, ensure contact information for essential follow-ups in place, and address other pain points surrounding the recruitment and enrollment processes.

#### Virtual messaging

Virtual messaging consists of technology-based individual communications, primarily in formats such as email or SMS messaging. Two researchers compared email to direct physical mailing in the recruitment of participants to a clinical trial. Following an iterative process, the optimized email campaign showed an increase in enrollment rate.^30,37^ The optimized email campaign was personalized (including the recipient's name) and focused primarily on access to new technologies in the subject line. The direct mailing campaign had a similar success rate to the original email.

Stanton et al. studied various recruitment strategies during a 33-month period for three distinct projects.^2^ Call-to-action emails from the Avon Army of Women (launched in 2008 to recruit women to participate in clinical research) were the most successful and resulted in increases in enrollment over time (they did not participate in recruitment for the first project), demonstrating much higher rates of enrollment over pay-per-click advertising or traditional recruitment flyers/print.

The speed of identifying recruits for the acute treatment of medical conditions is another area of opportunity identified in the literature for the use of virtual messaging digital technology in clinical trial recruitment. It is reported that only 1/3 of acute treatment trials reach their targeted number of patients within the planned timeframe due to identification delays. One study explored the use of a standardized algorithm, combined with a text message to physicians’ mobile phones, to quickly identify potentially eligible acute stroke patients for a clinical trial.^44^ In this case, a text message was correctly sent to 96.6% of eligible patients with a median time of 62 min from admission to a text message. Results suggested a strong use case for machine learning and data mining, combined with virtual communication, to be used for clinical trial recruitment, as it can be used outside normal working hours, is low in cost, and can be integrated into an existing emergency room process which may allow for a near-immediate identification of potential participants depending on the condition being studied.

## Discussion

This study identified 30 articles (21 in main results and nine additional commentary articles in discussion of subthemes) published in the interdisciplinary academic literature focused on utilizing different forms of digital technology to enhance recruitment and enrollment in clinical trials. These studies were primarily indexed in PubMed and describe and report how digital technologies appear to offer several advantages over traditional methods for improving recruitment and enrollment for clinical trials. However, they also highlight specific challenges that may limit their potential implementation and more wide-scale use.

Advantages these digital technologies provide to optimize clinical trial recruitment and enrollment were several, including using digital technology to expand the reach of clinical trials, reducing the need for physical research settings, improving the efficiency and convenience of clinical trial recruitment and enrollment through improved communication, increasing access to a greater number of potential participants, and enhancing data collection.^3,9,30,34,45^ However, there were also noted disadvantages. For example, participants who use digital health technologies are generally in younger populations. Hence, there appears to be a level of technology literacy required for the effective use of digital technology to recruit and enroll participants in clinical trials, and this may limit participation from groups with lower educational attainment or those who lack access to devices and Internet and mobile connectivity.^40^ There are also increased requirements relating to upfront financial costs and time for learning and implementation, thereby potentially causing hesitancy toward the adoption of these new technologies.

Multiple articles also discussed the opportunity for the use of digital technology to expand the reach of clinical trials, while also emphasizing the ability to reduce the need for physical research settings which may hinder access to participants, such as in the case of distributed or decentralized trial designs (e.g. trials that use “virtual” tools to deliver and administer clinical trial recruitment, medication delivery, and acquisition of trial outcomes without involving in-person contact between study teams and patients/subjects).^9,10,30,34,45^ However, this was also punctuated by concerns regarding the validation of technologies for use in a clinical research setting.^23^

There also appears to be an upward longitudinal trend of published literature on the topic of digital technologies in clinical trial recruitment and enrollment. The majority of studies which met study inclusion criteria were published in 2022 (four total), though searches were conducted only through July 2022. However, none of the included literature discussed the application of digital technology during the enrollment or recruitment phases of Phases I–III drug development trials and the majority of the original research (59%, *n* = 10) only involved data analysis for observational studies, with no evidence relating to the influence of randomization on the effectiveness of digital technology for recruitment or enrollment.

Globally, there appears to be a lack of extensive discussion regarding the application of digital technology in improving ethnic and racial minority recruitment and enrollment in clinical trials. This may be partly driven by the lack of reporting or data collection on race and ethnicity in studies examined. There is still extensive research that needs to be conducted to explore the effectiveness of current and emerging recruitment and enrollment tools for these hard-to-research populations. Less than one-fifth of the included literature explored minority recruitment or enrollment in any meaningful way. Policymakers and regulators may be aware of these challenges, with race and ethnicity data capture and reporting now required in new drug applications by the FDA and recently the FDA announced draft guidance to the industry to encourage the development of plans to enroll more participants from underrepresented racial and ethnic populations, underscoring increased attention to this issue.

The literature reviewed also suggested a lack of regulatory guidance regarding the use of digital technologies in clinical trials, specifically in the areas of recruitment and enrollment. E-consenting appears to have been used effectively for several years, primarily since extensive guidance regarding this specific use of technology has come from regulatory bodies. However, other digital technologies are yet to have specific regulations set forth, and this lack of oversight may lead researchers, trial sites, and study sponsors to hesitate in using available digital technologies out of concern that regulators and external monitoring boards, including Institutional Review Boards (IRBs), might delay digitally enabled research activities. This may partly explain the lack of studies examining the use of digital technology in recruitment or enrollment for drug development trials. The drug development process is highly regulated and the introduction of new or unproven technologies could potentially delay trial recruitment or otherwise negatively impact a regulatory submission supporting a drug dossier. Presumably, a lack of evidence supporting digital technologies’ effectiveness may further dampen regulatory support and subsequently industry adoption along with ongoing concerns about privacy considerations (see Supplemental File for more information).

We were also concerned that our literature review did not accurately reflect the real-world commercialization of software applications for the clinical trial space. In order to further investigate the general availability of software applications that might be commercially available, we conducted a search of Google Play and the Apple App Store in August 2022 to assess what mobile applications might be publicly available for clinical trial-related recruitment or enrollment. Based on this search, authors were only able to identify 14 apps between the first 20 results using the “clinical trials” keyword on both platforms (six on Google Play, seven on Apple App Store, and one that was available on both platforms) that actively involved recruitment or enrollment. Of those apps, the download numbers on the Google Play platforms ranged from <100 to 10,000 downloads, and the number of reviews for the Apple apps had a maximum of 15 across all eight apps. This suggests that consumer and commercial adoption of mobile applications for clinical trial recruitment and enrollment is still not widespread.

Another observation from the literature was that digital technologies for recruitment in clinical trials may be especially helpful due to the challenges associated with the COVID-19 pandemic. Traditional recruitment strategies have known limitations, which may have been exacerbated by the pandemic.^10^ For example, reduced foot traffic to clinics and hospitals limits the effectiveness of onsite recruitment. Advertisements on public transportation or print media may not receive as much attention as fewer people engage in traditional routines like commuting to school and work. With digital technologies, recruitment for clinical trials can expand across states, as participants may not need to be limited by geographic location. This may facilitate increased diversity of participants and inclusion of rural residents who may not have easy access to research sites that are concentrated in urban settings. Continued industry adoption of distributed or decentralized trials may further accelerate digital tool adoption for recruitment and enrollment, and may also be influenced by challenges experienced during COVID-19.

### Other subthemes specific to health equity and clinical trials

Fundamental challenges associated with clinical trial diversity and inclusivity were also highlighted in the literature reviewed and deserve special attention. For example, certain social determinants of health including socioeconomic status, demographics, and health literacy were reported to impact the ability of an individual to engage with digital components of clinical trials.^34^ The hindrance of access to electronic platforms due to sociodemographic, gender and geographic barriers is known as the digital divide.^44^ Limited awareness, discomfort, differences in typical healthcare utilization and search habits, lack of understanding, and distance to a health facility were cited as the main reasons for not using devices to access digital health.^44^ There is some speculation in the literature that the use of technology to recruit participants may exacerbate this digital divide bias, wherein some disadvantaged populations such as the elderly, disabled, or impoverished have less access to technology for recruitment and enrollment.^34^ As lower health literacy is more prevalent in non-White populations, digital health tools that do not adequately address disparities in technology and health literacy may exacerbate the digital divide.^46^ However, it has also been suggested that this divide continues to shrink as greater portions of the U.S. population have gained access to reliable internet, smartphones, computers, and other technologies.^3,5^

Researchers also assert that digital recruitment may allow researchers to connect with marginalized individuals who live in rural areas and are unable to travel to recruitment sites, allowing for broader recruitment criteria and higher external validity.^34^ Van Norman discussed how crowdsourcing through an online community for recruitment of participants for an HIV clinical trial resulted in greater participation from women, racial and ethnic minorities, and low-income individuals when compared to recruiting through traditional community advisory boards.^34^ Another study also demonstrated that the use of an audiocard geared toward African American women had a statistically significant impact on assessments of personal value, social influence and personal barriers with regard to clinical trials but did not impact behavioral intent to enroll.^36^

A study conducted by Banda et al. in the multimedia presentation technology category utilized a culturally targeted video intervention to increase the participation of African American patients in their cancer clinical trials.^47^ A community advisory group guided the development of video content consisting of African American patients discussing their lived experiences with clinical trials. Members of the clergy, family members, and medical personnel were also included in the unscripted video. A total of 111 patients completed a questionnaire both before and after watching the intervention video, with the post-questionnaire asking about intent to enroll. The proportion of patients expressing the likelihood to enroll in a clinical trial increased, from 45.9% pre-video to 79.6% post-video. The results of the study demonstrated the potential positive influence of a multimedia presentation to address attitudinal barriers specific to certain minority populations for participation in trials.

It was also reported that attitudinal barriers continue to be a concern for the recruitment and successful enrollment of racial and ethnic minorities into clinical trials. Researchers cataloged some of these barriers specific to ethnic and minority populations including (1) fear and distrust of the medical establishment, (2) concerns regarding loss of autonomy, (3) worry over mistreatment, (4) concern about the ethical conduct of investigators, (5) privacy concerns, and (6) lack of knowledge and awareness regarding trials.^29,45^ Furthermore studies found that African American women have had greater skepticism toward research than European American women and are less likely to participate in clinical trials. Gonzalez and Grov discuss how women of color may be more willing to be recruited through known and trusted adults rather than through anonymous banner ads, which they demonstrated in their randomized two-group pilot study, further illustrating challenges associated with attitudinal barriers and digital recruitment tools.^37^

### Limitations

Future studies should assess a broader base of literature around randomized clinical trials that assess the utility of digital technologies as these studies become more available. Additionally, the search parameters of our methods may have limited the results due to the use of terms such as “clinical trials” which may have produced only studies that reference themselves in the abstract and/or title as a trial as opposed to a study. Additionally, it may be valuable to limit the scope of a review on this topic to a specific disease or condition (e.g., cancer, which was our most frequently observed disease/condition noted in the literature) as the impact of each technology may be dependent on individual disease and less generalizable than for what this study could account for.

## Conclusion

Our systematic review of the literature identified support for the potential use of several digital technologies to increase the effectiveness of recruitment and enrollment for clinical trials. However, research around this topic is still emerging, and many of the articles did not provide empirical data supporting technology use and instead came in the form of opinions or commentaries and applications used in observational studies. The opportunities for digital technology to improve efficiencies, including cost-effectiveness, are highly speculated in available research, but there appears to be no consensus in the literature, particularly when considering the diversity of technology types, different types of trials, and various diseases and conditions that may benefit in different ways from the digital optimization of trial recruitment and enrollment. Further, there is little evidence suggesting the purposeful use of these digital technologies to enhance shared health equity goals, specifically for addressing needed improved enrollment and recruitment of racial and ethnic minorities and other difficult-to-reach populations. Additional research, industry commitment, and regulatory guidance are needed across multiple disease types and various digital technologies to determine the best approaches for leveraging these technologies to promote trial diversity and equity, lower costs, improve throughput, and reach enrollment goals.

## Supplemental Material

sj-docx-1-dhj-10.1177_20552076241242390 - Supplemental material for Digital technologies used in clinical trial recruitment and enrollment including application to trial diversity and inclusion: A systematic review Supplemental material, sj-docx-1-dhj-10.1177_20552076241242390 for Digital technologies used in clinical trial recruitment and enrollment including application to trial diversity and inclusion: A systematic review by Amy Kasahara, Jennifer Mitchell, Joshua Yang, Raphael E. Cuomo, Tiana J. McMann and Tim K. Mackey in DIGITAL HEALTH

sj-docx-2-dhj-10.1177_20552076241242390 - Supplemental material for Digital technologies used in clinical trial recruitment and enrollment including application to trial diversity and inclusion: A systematic review Supplemental material, sj-docx-2-dhj-10.1177_20552076241242390 for Digital technologies used in clinical trial recruitment and enrollment including application to trial diversity and inclusion: A systematic review by Amy Kasahara, Jennifer Mitchell, Joshua Yang, Raphael E. Cuomo, Tiana J. McMann and Tim K. Mackey in DIGITAL HEALTH
