# Supplementary material for: Digital technologies used in clinical trial recruitment and enrollment including application to trial diversity and inclusion: A systematic review
Source: Digit Health. 2024 Mar 28;10:20552076241242390. doi: 10.1177/20552076241242390 (PMC10981266; doi:10.1177/20552076241242390)
Supplement: sj-docx-2-dhj-10.1177_20552076241242390 - Supplemental material for Digital technologies used in clinical trial recruitment and enrollment including application to trial diversity and inclusion: A systematic review [file sj-docx-2-dhj-10.1177_20552076241242390.docx]

**Supplementary File**

**METHODS**

*Details on MeSH and Keywords used for Systematic Review*:

The three databases were searched for articles published in the English language between January 2012 and July 2022, using the Medical Subject Headings (MeSH) unique ID terms “Clinical Trials” (ID: D002986) and “Technology” (ID: D013672). Other non-MeSH key search terms included “Clinical Trials,” “Technology,” “Enroll*,” “Recruit*,” and “Digital”. Keywords were queried in the Title/Abstract field using the advanced search function settings for PubMed, IEEE Xplore, and ACM Digital Library databases.

In PubMed our search parameters consisted of ("Clinical Trials as Topic"[Mesh]) AND "Technology"[Mesh] AND (enroll* OR recruit*) and “Clinical Trials AND Technology AND Digital” found in abstract/title which provided 411 results. In IEEE Xplore we used the search parameters “Clinical Trials AND Technology AND (enroll* OR recruit*)” and “Clinical Trials AND Technology AND digital” found in abstracts which produced 12 results. In ACM Digital Library our search parameters included “Clinical Trials AND Technology AND (enroll* OR recruit*)” and “Clinical Trials AND Technology AND digital” found in abstracts which provided 190 results (see **Appendix Table 1**).

**Appendix Table 1: Search Parameters by Source**

| **PubMed Search (Abstract/Title)** | **All Years** | **2012-2022** |
| --- | --- | --- |
| ("Clinical Trials as Topic"[Mesh]) AND "Technology"[Mesh] AND (enroll* OR recruit*) | 227 | 136 |
| Clinical Trials AND Technology AND digital | 318 | 275 |
| Total: | 545 | 411 |
| **IEEE Xplore (Abstract)** | **All Years** | **2012-2022** |
| Clinical Trials AND Technology AND (enroll* OR recruit*) | 0 | 0 |
| Clinical Trials AND Technology AND digital | 23 | 12 |
| Total: | 23 | 12 |
| **ACM Digital Library (Abstract)** | **All Years** | **2012-2022** |
| Clinical Trials AND Technology AND (enroll* OR recruit*) | 37 | 21 |
| Clinical Trials AND Technology AND digital | 282 | 169 |
| Total: | 319 | 190 |

2022*: Data was pulled on July 20, 2022

*Additional PRISMA Protocol and Methods*

**2.1 Study selection**

To minimize errors and reduce potential biases in study selection, first and second authors, AK and JM, independently applied inclusion and exclusion criteria that filtered results by initially reviewing abstracts and, subsequently, full text of all extracted articles. Any discrepancies regarding study eligibility were resolved through discussion among the authors to reach a final consensus. Articles included in qualitative synthesis were reviewed and categorized by technology type to determine which, if any, category of technology is utilized in the clinical trial recruitment/enrollment. The calculation of the Cohen's Kappa statistic was undertaken to assess the level of concordance between the evaluators. In this calculation, the total instances or observations correspond to the aggregate count of abstracts and complete articles initially discovered during the search phase. Concordant observations were the count of articles where both raters agreed in their decision about inclusion or exclusion, adhering to the established criteria. Based on this methodology, we report a Cohen’s Kappa of 0.73, indicating good inter-coder agreement.

**2.2 Inclusion criteria**

Articles were included in this review if they discussed or examined online technology in the context of clinical trials enrollment and/or recruitment, including articles addressing privacy or the use of these tools for enrollment of racial and ethnic minority populations or lower socioeconomic status groups.

**2.3 Exclusion criteria**

Initially, articles were excluded if they did not discuss the application of technology in the clinical trial recruitment or enrollment process (e.g., discussed other uses of technology in clinical trials). Conversely, articles that included clinical trial recruitment/enrollment but did not discuss forms of technologies (e.g., non-digital and solely field-based solutions) were excluded. We did not exclude literature based on specific content types or study designs.

**2.4 Bias assessment**

If any randomized controlled trials (RCTs) assessing trial recruitment as a primary endpoint were selected for inclusion, the Cochrane Collaboration’s tool for assessing risk of bias was used. The influence of publication bias should be assessed vis-à-vis a funnel plot in the case that comparable quantitative measures were identified across studies. For all other types of studies, the influence of biases was qualitatively evaluated by the authors, which is a technique previously employed in systematic reviews in the area of health policy research.

**RESULTS**

*Geography, technology type, and number of publications by year*:

The majority of studies were from the United States (76.7%) with one study each published from co-authors in Switzerland, Canada, Germany, England, and Australia; and two studies that involved the collaboration of multiple countries (United States, China, Taiwan, Singapore, United Kingdom, Australia). We also observed an upward trend in publications over time that met our inclusion criteria (see **Appendix Table 2** for geography and number of publications by year).

**Appendix Table 2. Descriptive Statistics of Themes and all Subthemes**

| **Theme** | **Subcategory** | **# of Articles** | **% of Total*** |
| --- | --- | --- | --- |
| *Technology Type** |  |  |  |
|  | Blockchain | 2 | 7% |
|  | Mobile Apps | 4 | 13% |
|  | Machine Learning/EHR | 7 | 23% |
|  | Multi-media | 4 | 13% |
|  | Web Platform | 9 | 30% |
|  | E-Consent | 5 | 17% |
|  | Virtual Messaging | 8 | 27% |
|  | Social Media | 10 | 33% |
| *Geography* |  |  |  |
|  | United States | 23 | 77% |
|  | Australia | 1 | 3% |
|  | Canada | 1 | 3% |
|  | England | 1 | 3% |
|  | Germany | 1 | 3% |
|  | Switzerland | 1 | 3% |
|  | Multinational | 2 | 7% |
| *Year of Publication* |  |  |  |
|  | 2012 | 2 | 7% |
|  | 2013 | 2 | 7% |
|  | 2014 | 1 | 3% |
|  | 2015 | 3 | 10% |
|  | 2016 | 0 | 0% |
|  | 2017 | 2 | 7% |
|  | 2018 | 3 | 10% |
|  | 2019 | 2 | 7% |
|  | 2020 | 4 | 13% |
|  | 2021 | 5 | 17% |
|  | 2022^a^ (partial year) | 6 | 20% |
| *Diseases Studied* |  |  |  |
|  | Cancer | 8 | 27% |
|  | Cardiovascular | 1 | 3% |
|  | COVID19 | 1 | 3% |
|  | Lifestyle/Risk Factors | 5 | 17% |
|  | Neuro | 4 | 13% |
|  | Not Specified | 11 | 37% |
| *Ethnic and Minority Considerations* |  | 11 | 37% |
| *Privacy & Regulatory* |  | 6 | 20% |
| *Cost/Benefit Analysis* |  | 8 | 27% |

*Some articles explored more than one subcategory so totals may not be calculating to 100% and the % of total describes the number of articles that each subtheme was included in out of the total number of articles included in this review.

^a^ Indicates number of articles that met inclusion criteria for this study but only for a partial year of 2022 (up to July 2022)

*Geography of Publications (Appendix Figure 1)*:


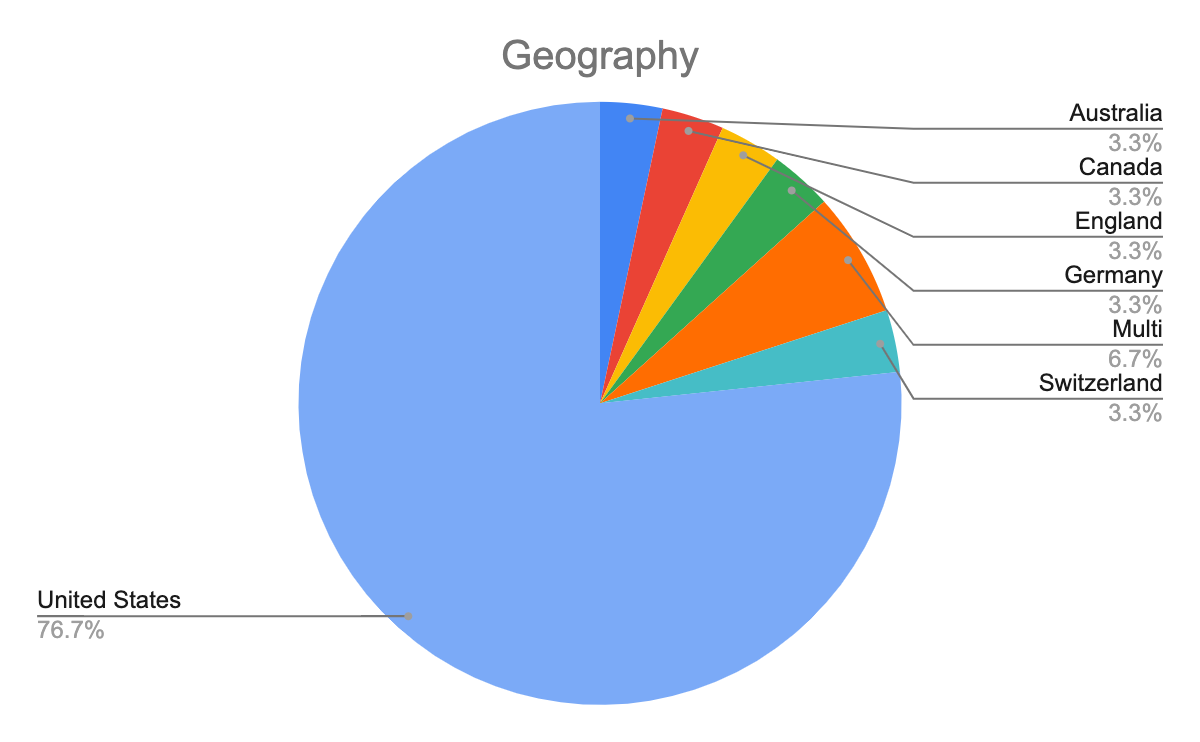


*Findings from Randomized Controlled Studies*:

Four randomized controlled trials (RCTs) met study inclusion criteria. The technology types for the RCTs included the use of web-based materials, namely, QR codes^28^, multimedia technology through the use of an interactive iPad program^29^, virtual messaging via optimized emails^30^, and machine learning to automate eligibility notification, delivery of research materials, and informed consent.^31^ The use of optimized emails found a 12x increase in participant enrollment compared to the baseline email invitation.^30^ The use of web-based materials featured a combination of traditional and contemporary methods (print materials, online ads, social media, QR codes), all of which led to a study website and online form for prospective participants and found that recruitment of individuals through an engaging Internet facilitated method aided with retention and interaction with trial interventions and is cost-effective and scalable compared to non-digital materials.^28^ The use of machine learning during the recruitment and enrollment phases of trials found that while this modality was not solely effective in enrollment, its use was successful in the identification, randomization, and collection of electronic signatures of participants.^31^ Multimedia use showed increased comprehension in clinical trial concepts among children and a 67% favorability over traditional paper format.^29^

*Cost/Benefit Results*

The comparison of costs and benefits was addressed in the literature in the context of digital tools. Many articles suggested that use of digital technologies can reduce costs related to recruitment and enrollment.^3,9,30,33,34^ For example, in one study, the average cost per click for Facebook recruitment averaged $.17 to $.69 and totaled $120,000, though they assert that these costs are lower compared to the cost of multiple sites and personnel salaries necessary to recruit hard-to-reach populations with sufficient sample size.^20^ However, in contrast, a total of $704.75 was spent on Facebook ads and $287 was spent on OkCupid ads for a study, resulting in only 2 enrolled subjects from Facebook ($352/participant) and 0 enrollees from OkCupid.^37^ These were both substantially higher costs than the $175 spent on targeted electronic recruitment which resulted in 110 participants ($1.59/participant).

One study demonstrated that their automated pre-screening computer-based algorithm was cost-neutral ($4,123 with chart review and $4,116 with prescreening algorithm) after enrolling 12 subjects.^42^ They projected that the cost benefits of the algorithm would grow with more participants, estimating $17,181 for 50 enrollees with chart review and only $8,437 using the algorithm.

Overall, there were inconsistent findings regarding the financial benefit of the use of digital technology for enrollment and recruitment, with some digital technologies (e.g., email distribution) being viewed as more cost effective to reach large populations when compared to other technologies (e.g. social media ads). This lack of consensus may stem from an absence of standards regarding calculations of cost, such as cost per click versus cost per enrollee and an overall low number of studies published in the literature. Due to the lack of consensus, an agreed upon or standardized metric might provide an opportunity to compare cost effectiveness of digital technologies with traditional methods for clinical trial enrollment and recruitment.

*Additional privacy and regulatory considerations*

While digital technologies utilized in clinical trials offer many opportunities for enhancing recruitment, enrollment, and other clinical trial management practices, they also create vulnerabilities for breach of data and concerns related to privacy.^1,10,47^ As technology advances, there is an evolving need for responsible data processes surrounding privacy, security, and the transparency of these processes.^44^

One area of potential concern is the use of mobile phones in clinical trials.^47,44^ Mobile phones are commonly used to send messages to participants in clinical trials which may inadvertently disclose disease status. Additionally, mobile phones with location services can record the location and use of apps and connected devices, which may be used in clinical protocols. This data can potentially be shared downstream with other apps or databases. Andriesen et al. discuss the risks present when participants use their personal devices for study communications and suggest one solution to this privacy risk may be the use of a personal identification number (PIN) to trigger a text message with trial-specific content.^47^ Mitigations such as this will need to be explored to ensure privacy for potential participants.

Patient and participant privacy is a key concern in digital approaches in research and community engagement. Vulnerabilities may arise in communities, particularly lower socioeconomic groups, who face obstacles in negotiating digital privacy and confidentiality. Privacy concerns may arise in digital crowdsourcing and qualitative approaches, but these risks can be mitigated by limiting the identifying information collected, not requiring real names, allowing participants to opt-out of specific questions, and co-creating study protocols.^34^ Researchers discussed how regulatory frameworks may require further review to ensure protocols align with privacy and safety in digital approaches, including those that govern recruitment and enrollment practices.^34^ It has been recommended that regulators and committees should provide additional recommendations and guidance regarding risks and benefits of digital approaches as this area continues to evolve.^34^

*Potential Bias in Study Findings*

RCTs with primary endpoints as trial recruitment were not uncovered in the extant literature on the topic of the use of digital technologies for enrollment and recruitment in clinical trials, so the Cochrane Collaboration tool for risk of bias in RCTs was not applied. Similarly, a funnel plot was not able to be created due to lack of comparable quantitative effect estimates across studies. Subjectively, the risk of publication bias appears low due to the discovery of studies showing both positive and null effects from the use of digital technologies for trial enrollment and recruitment. However, results of studies may be biased due to convenience and familiarity of investigators with the studied digital technologies. In particular, lack of familiarity with newer (and potentially more impactful) forms of digital technology for the purpose of enrollment/recruitment may bias study results toward the null.

*Full List of Articles Reviewed and Included (see Appendix Table 3 below)*

**Appendix Table 3: Full List of Systematic Review Results (including both original research, reviews, and non-original research articles)**

| **Title** | **Authors** | **Year** | **Original Research (Y/N)** | **Content Type** | **Category of Tech** | **Disease or general** | **Disease category** | **Privacy sub-topic** | **Geography** |
| --- | --- | --- | --- | --- | --- | --- | --- | --- | --- |
| [Using Digital Technologies in Clinical HIV Research: Real-World Applications and Considerations for Future Work](https://pubmed.ncbi.nlm.nih.gov/28760729/) | Andriesen J | 2017 | N | Commentary | Virtual Messaging, Multi-media | HIV | Lifestyle/Risk Factors | Yes | United States |
| [Assessment of automated clinical trial recruitment and enrolment using patient-facing technology](https://pubmed.ncbi.nlm.nih.gov/33504589/) | Bardach, N | 2021 | Y | Original Research - RCT | Machine Learning | Not Specified | Not Specified | No | United States |
| [Automated real-time text messaging as a means for rapidly identifying acute stroke patients for clinical trials](https://pubmed.ncbi.nlm.nih.gov/25073719/) | Jegzentis, K | 2014 | Y | Original Research - observational | Virtual Messaging, Machine Learning | Stroke | Neuro | No | Germany |
| [Deployment of an End-to-End Remote, Digitalized Protocol in COVID-19: Process Evaluation](https://pubmed.ncbi.nlm.nih.gov/35852933/) | Zahradka, N | 2022 | Y | Original Research - observational | Social Media, E-consent | COVID 19 | COVID-19 | No | United States |
| [Development of a Digital Research Assistant for the Management of Patients' Enrollment in Oncology Clinical Trials within a Research Hospital](https://pubmed.ncbi.nlm.nih.gov/33801668/) | Cesario A | 2021 | Y | Original Research - observational | Mobile Applications, Machine Learning | Cancer | Cancer | No | Switzerland |
| [Digital recruitment and enrollment in a remote nationwide trial of screening for undiagnosed atrial fibrillation: Lessons from the randomized, controlled mSToPS trial](https://pubmed.ncbi.nlm.nih.gov/30656241/) | Baca-Motes K | 2019 | Y | Original Research - RCT | Virtual Messaging | Atrial Fibrillation (for stroke prevention) | Cardiovascular | No | United States |
| [Digital technologies as biomarkers, clinical outcomes assessment, and recruitment tools in Alzheimer's disease clinical trials.](https://pubmed.ncbi.nlm.nih.gov/29955666/) | Gold M, Amatniek J | 2018 | N | Commentary | Web platform, Social Media | Alzheimer's | Neuro | No | United States |
| [Digitizing clinical trials.](https://pubmed.ncbi.nlm.nih.gov/32821856/) | Inan OT | 2020 | N | Commentary | Social Media, E-consent, Virtual Messaging, Machine Learning | Not Specified | Not Specified | No | United States |
| [Efficacy and cost-effectiveness of an automated screening algorithm in an inpatient clinical trial](https://pubmed.ncbi.nlm.nih.gov/22308560/) | Beauharnais, C | 2012 | Y | Original Research - observational | Machine Learning | Diabetes | Lifestyle/Risk Factors | No | United States |
| [Facebook Recruitment for Children with Advanced Cancer and Their Parents: Lessons from a Web-based Pediatric Palliative Intervention Study](https://pubmed.ncbi.nlm.nih.gov/34737490/) | Cho E | 2021 | Y | Original Research - observational | Social Media, | Pediatric Palliative Cancer | Cancer | No | United States |
| [Improving the Patient-Clinician Interface of Clinical Trials through Health Informatics Technologies](https://pubmed.ncbi.nlm.nih.gov/29845581/) | Carrion, J | 2018 | N | Brief Review | Social Media, Machine Learning, Mobile Applications | Not Specified | Not Specified | No | United States |
| [Incorporating Digital Tools to Improve Clinical Trial Infrastructure: A White Paper From the Digital Engagement Committee of SWOG](https://pubmed.ncbi.nlm.nih.gov/30652537/) | Dizon DS | 2018 | N | Commentary | Multi-media | Cancer | Cancer | No | United States |
| [Innovating information-delivery for potential clinical trials participants. What do patients want from multi-media resources?](https://pubmed.ncbi.nlm.nih.gov/22819269/) | Shneerson, C | 2013 | Y | Original Research - observational | Multi-media, Web platform | Not Specified | Not Specified | No | England |
| [Responding to a significant recruitment challenge within three nationwide psychoeducational trials for cancer patients](https://pubmed.ncbi.nlm.nih.gov/23595235/) | Stanton, A | 2013 | Y | Original Research - observational | Virtual Messaging, Web Program | Cancer | Cancer | No | United States |
| [Using digital multimedia to improve parents' and children's understanding of clinical trials.](https://pubmed.ncbi.nlm.nih.gov/25829422/) | Tait AR | 2015 | Y | Original Research - RCT | Multi-media | Not Specified | Not Specified | No | United States |
| [Using e-technologies in clinical trials.](https://pubmed.ncbi.nlm.nih.gov/26176884/) | Rosa C | 2015 | N | Commentary | Social Media, Vitual Messaging, Web platform, Mobile Apps, Machine Learning, E-consenting | Not Specified | Not Specified | No | United States |
| [Artificial Intelligence for Clinical Trial Design](https://ieeexplore.ieee.org/document/9353614/) | Stefan Harrer | 2020 | N | Commentary | Machine Learning | Neurology | Neuro | Yes | Australia |
| [Re-engineering a Clinical Trial Management System Using Blockchain Technology: System Design, Development, and Case Studies](https://pubmed.ncbi.nlm.nih.gov/35759315/) | Zhuang, Y | 2022 | N | Commentary | Blockchain | General | Not Specified | No | Multi |
| [Clinical trial management of participant recruitment, enrollment, engagement, and retention in the SMART study using a Marketing and Information Technology (MARKIT) model](https://pubmed.ncbi.nlm.nih.gov/25866383/) | Gupta A | 2015 | Y | Original Research - RCT | Social Media, Virtual Messaging, Web platform | weight loss | Lifestyle/Risk Factors | Yes | United States |
| [Association of Remote Technology Use and Other Decentralization Tools With Patient Likelihood to Enroll in Cancer Clinical Trials](https://pubmed.ncbi.nlm.nih.gov/35788672/) | Adams, D | 2022 | Y | Original Research - observational | E-consenting, Mobile Applications | Cancer | Cancer | No | United States |
| [Digital Clinical Trials for Substance Use Disorders in the Age of Covid-19.](https://pubmed.ncbi.nlm.nih.gov/32956164/) | Brezing CA | 2020 | N | Commentary | Web platform, Social Media | SUD | Lifestyle/Risk Factors | No | United States |
| [Digital Health Applications for Pharmacogenetic Clinical Trials.](https://pubmed.ncbi.nlm.nih.gov/33114567/) | Naik H | 2020 | N | Brief Review | Web platform, E-consent, Machine Learning | General | Not Specified | No | United States |
| [Social Media Use for Research Participant Recruitment: Integrative Literature Review](https://www.jmir.org/2022/8/e38015) | Darko, E | 2022 | N | Brief Review | Social Media | General | Not Specified | Yes | Canada |
| [A pilot study of a culturally targeted video intervention to increase participation of African American patients in cancer clinical trials](https://pubmed.ncbi.nlm.nih.gov/22639112/) | Banda, D | 2012 | Y | Original Research - pilot study | Multimedia Presentation | Cancer | Cancer | No | United States |
| [Investigation of a multimedia, computer-based approach to improve knowledge, attitudes, self-efficacy, and receptivity to cancer clinical trials among newly diagnosed patients with diverse health literacy skills](https://pubmed.ncbi.nlm.nih.gov/30811591/) | Polite, B | 2019 | Y | Original Research - observational | Multi-media | Cancer | Cancer | No | United States |
| [Processes in Increasing Participation of African American Women in Cancer Prevention Trials: Development and Pretesting of an Audio-Card](https://pubmed.ncbi.nlm.nih.gov/29131708/) | Kenerson, D | 2017 | Y | Original Research - RCT | Multi-media | Cancer | Cancer | No | United States |
| [Recruiting young women of color into a pilot RCT targeting sexual health: Lessons learned and implications for applied health technology research](https://pubmed.ncbi.nlm.nih.gov/32343193/) | Gonzalez, S | 2022 | Y | Original Research - observational | Social Media, Virtual Messaging | Sexual Health | Lifestyle/Risk Factors | No | United States |
| [Digital approaches to enhancing community engagement in clinical trials](https://pubmed.ncbi.nlm.nih.gov/35338241/) | Tan RKJ | 2022 | N | Commentary | Web platform | Not Specified | Not Specified | Yes | Multi |
| [Decentralized Clinical Trials](https://www.ncbi.nlm.nih.gov/pmc/articles/PMC8093545/) | Van Norman, G. | 2021 | N | Brief Review | Web platform, blockchain | General | Not Specified | Yes | United States |
| General Practice and Digital Methods to Recruit Stroke Survivors to a Clinical Mobility Study: Comparative Analysis | Reuter, K | 2021 | Y | Original Research - observational | Social media, web platform, | Stroke | Neuro | No | United States |
